# Association Between Early Immunosuppression Center Variability and One‐Year Outcomes After Pediatric Liver Transplant

**DOI:** 10.1111/petr.70018

**Published:** 2025-01-08

**Authors:** Vikram K. Raghu, Scott D. Rothenberger, James E. Squires, Elizabeth Eisenberg, Anna L. Peters, Jennifer Halma, Swati Antala, Irini D. Batsis, Ke‐You Zhang, Amy G. Feldman, Daniel H. Leung, Steven J. Lobritto, John Bucuvalas, Simon P. Horslen, George V. Mazariegos, Emily R. Perito

**Affiliations:** ^1^ Department of Pediatrics University of Pittsburgh School of Medicine and UPMC Children's Hospital of Pittsburgh Pittsburgh Pennsylvania USA; ^2^ Department of Medicine University of Pittsburgh School of Medicine Pittsburgh Pennsylvania USA; ^3^ Patient and Family Voice Starzl Network for Excellence in Pediatric Transplantation Pittsburgh Pennsylvania USA; ^4^ Department of Pediatrics University of Cincinnati College of Medicine Cincinnati Ohio USA; ^5^ Division of Pediatric Gastroenterology, Hepatology and Nutrition Cincinnati Children's Hospital Medical Center Cincinnati Ohio USA; ^6^ Division of Pediatric Gastroenterology Children's Mercy Kansas City Kansas City Missouri USA; ^7^ Department of Pediatrics, Mount Sinai Kravis Children's Hospital Icahn School of Medicine at Mount Sinai Hospital New York New York USA; ^8^ Department of Pediatrics Stanford University School of Medicine Stanford California USA; ^9^ Department of Pediatrics University of Colorado School of Medicine and Children's Hospital Colorado Aurora Colorado USA; ^10^ Division of Pediatric Gastroenterology, Hepatology and Nutrition, Department of Pediatrics Baylor College of Medicine Houston Texas USA; ^11^ Division of Pediatric Gastroenterology, Hepatology and Nutrition Columbia University Irving Medical Center, Morgan Stanley Children's Hospital New York New York USA; ^12^ Thomas E. Starzl Transplantation Institute Pittsburgh Pennsylvania USA; ^13^ Division of Pediatric Gastroenterology, Hepatology and Nutrition, Department of Pediatrics University of California San Francisco San Francisco California USA

**Keywords:** anti‐thymocyte globulin, basiliximab, corticosteroids, mycophenolate mofetil, t‐cell depleting antibody

## Abstract

**Background:**

Despite the existence of institutional protocols, liver transplant centers often have variability in early immunosuppression practices. We aimed to measure within‐center variability in early immunosuppression after pediatric liver transplant (LT) and examine its association with one‐year outcomes.

**Methods:**

We analyzed pediatric LTs from 2013 to 2018 in the United Network for Organ Sharing registry, with data aggregated by center. We categorized induction regimen as corticosteroids only vs. T‐cell depleting antibody vs. non‐T‐cell depleting antibody. Primary exposures were coefficient of immunosuppression variability (CIV) in (1) induction and (2) mycophenolate mofetil (MMF) use. Primary outcomes were one‐year graft survival, patient survival, and acute rejection rate within the first year after transplant.

**Results:**

The study cohort included 2542 LT recipients from 67 LT centers. Sixteen centers (24%) had no MMF variability; twenty‐five centers (37%) had no induction variability. In multivariable analysis, induction CIV was associated with 2.72 times greater odds of acute rejection in the first year (OR 2.72; 95% CI 1.66–4.45; *p* < 0.001). MMF CIV was not associated with rejection (OR 1.22, 95% CI 0.66–2.25, *p* = 0.527). Neither one‐year graft nor patient survival were associated with induction or MMF CIV.

**Conclusions:**

Induction CIV is associated with higher one‐year acute rejection odds and did not impact short‐term graft or patient survival. Improved understanding of the reasons for high CIV will inform future work aiming to determine whether reducing variability may improve outcomes.

**Abbreviations**
CIV: coefficient of immunosuppression variability
MMF: mycophenolate mofetil
SNEPT: Starzl Network for Excellence in Pediatric Transplantation

## 1 Introduction

Early immunosuppression in liver transplantation plays a critical role in protecting the liver, yet little data exist supporting the optimal regimen. Retrospective data provide conflicting messages, even from large national database studies [1, 2]. As a result, centers are left to develop individual protocols based on anecdotal experience and historical practices. Despite all centers having immunosuppression protocols, variability frequently occurs, as demonstrated by a Society for Pediatric Liver Transplantation survey showing that at most centers protocol deviations were common [3]. The Starzl Network for Excellence in Pediatric Transplantation (SNEPT) has identified immunosuppression practice as a key area for optimization and established an immunosuppression project team to develop and execute stepwise approaches to identifying current practice and variation that would presage future work leading potentially to comparative effectiveness trials [4].

Induction immunosuppression can be classified into steroid therapy alone or one of two types of antibody induction: t‐cell depleting antibodies such as anti‐thymocyte globulin and non‐t‐cell depleting antibodies such as basiliximab. Maintenance regimens nearly always used tacrolimus but may include a decision of whether to use mycophenolate mofetil (MMF) as an additional agent. Complicating this, immunosuppression may be uniform across all patients at a center or may be individualized either by physician preference or in an attempt to personalize immunosuppression based on diagnosis or other patient characteristics. Ultimately, this personalization creates variability in immunosuppression practices at a center, and yet data are lacking to examine how this variability relates to patient outcomes. Our previous work examined the association between early immunosuppression choice and outcomes for individual patients [2]. However, as immunosuppression largely follows center practices, there is a critical need to understand how center‐based immunosuppression practices affect outcomes: Are we effectively personalizing immunosuppression to optimize outcomes or are we introducing unnecessary complexity with potentially increased side effects and costs?

To better understand this association, we must be able to measure variability. In this study, we introduce a coefficient of immunosuppression variability (CIV) based on the statistical concept of unalikeability, a method of characterizing categorical variable variability, to measure within‐center variability in immediate post‐transplant immunosuppression regimens [5]. Utilizing a novel approach of analyzing outcomes by center rather than by individual patient, we aimed to study for the first time the relationship between CIV and early outcomes after pediatric liver transplant.

## 2 Methods

We performed a retrospective analysis of prospectively collected data using the UNOS Standard Transplant Analysis and Research (STAR) files. This study was a secondary analysis of previously presented data analyzed at the individual level [2]. This study was given ethical approval by the University of California San Francisco Committee on Human Research (CHR# 20–30 171). A waiver of informed consent was granted. All research was conducted in accordance with both the Declarations of Helsinki and Istanbul.

### 2.1 Study Population

We identified children aged 0–18 who received a liver‐only transplant between 2013 and 2018 and had at least 1 year of post‐transplant follow‐up data available or died or lost their graft within the first year. We excluded children if they had received a previous non‐liver or concurrent multi‐organ transplant due to differences in IS choice and expected outcomes.

### 2.2 Study Variables

All study variables were aggregated at the level of the liver transplant center; the unit of analysis was the transplant center. The primary predictors of interest described immunosuppression within‐center variability. We classified individual patients by (1) induction regimen and (2) whether they received peri‐operative MMF. Induction regimen was defined as either corticosteroid alone, T‐cell depleting antibody with or without corticosteroids, or non‐T‐cell depleting antibody with or without corticosteroids. For induction regimen and MMF, CIV was calculated using the concept of unalikeability, which is a measure from the statistical literature of variability in categorical variables [5]. With continuous variables, we can determine variability using measures such as variance or standard deviation. With a categorical variable, standard deviation or variance would not be applicable. Instead, we must determine how alike a certain group may be, which gives rise to the concept of unalikeability or how unalike a group may be as a measure of variability. Greater unalikeability corresponds with more variability. The following equations were adapted from the existing literature on how to calculate unalikeability for a categorical variable [5]. For induction regimen, CIV was defined by the following equation:

$$\text{Induction}\;\mathrm{CIV}=1-p_{1}^{2}-p_{2}^{2}-p_{3}^{2}$$

where *p*_1_, *p*_2_, and *p*_3_ represent the proportion of individuals at a center that received corticosteroids only (*p*_1_), t‐cell depleting antibody (*p*_2_), and non‐T‐cell depleting antibody (*p*_3_), respectively. For MMF use with only two categories, the equation can be simplified to the following:

$$\mathrm{MMF}\;\mathrm{CIV}=2\times p\times \left(1-p\right)$$

where *p* is the proportion of individuals at a given center that received MMF.

The primary outcome measures were transplant center one‐year graft survival, one‐year patient survival, and incidence of rejection in the first year. Patient death was included as a graft loss event, as is the standard used by UNOS. Each outcome was reported as a proportion of patients that experienced the event at each center.

We considered additional variables in our analyses as possible confounders. Individual patient characteristics aggregated at the center level included sex, age, diagnosis, location at the time of transplant, and retransplant. Age was categorized as below 2 years, between 2–12 years, and 12–18 years. Diagnosis was categorized into biliary atresia, other cirrhotic disease, acute liver failure, tumor, and noncirrhotic disease, classified as previously reported [2]. Location was categorized into those at home, in the hospital, or in the intensive care unit immediately prior to transplant. Additionally, we defined a center‐level variable for center transplant volume with < 10 transplants/year, 10–20 transplants/year, or > 20 transplants/year. These variables were selected for the primary analysis because they were significant outcome predictors in the previous analysis performed at the individual level [2].

### 2.3 Statistical Analysis

Our primary analysis investigated associations of within‐center variability for (1) induction and (2) MMF use with one‐year graft survival, patient survival, and acute rejection. Center‐level data were summarized using descriptive statistics. Previous work summarized individual patient data for this cohort [2]. We performed multivariable binomial regression to determine the independent effect of CIV on center‐level outcomes. Univariate associations were determined with each predictor of interest. Predictors were included in multivariable backward selection with a significant univariate association (*p* < 0.20). For backward selection, the significance threshold was set at 0.05. It was a priori determined that both induction CIV and MMF CIV would be included in the final model to determine the independent effect of each variable. An interaction term was tested between induction CIV and MMF CIV in each model. Sensitivity analyses were performed for a model without necessarily including the two CIV terms as well as a model including only with centers with at least 5 transplants in the data set to exclude any confounding by small centers with an unstable estimate of both the primary predictor and primary outcomes.

## 3 Results

Our cohort included 67 pediatric liver transplant centers, with 2542 total pediatric liver transplant recipients included from those centers. Center volume ranged from 1 to 179 transplants over the study period. Fourteen centers performed five or fewer transplants over the study period; seven centers performed only one transplant over five years.

Table 1 summarizes center level characteristics of their transplant recipients. Centers largely transplanted a balanced cohort by sex. There was a slight predominance of recipients under 2 years old. Biliary atresia was the most common diagnosis, followed by other cirrhotic diseases. Centers transplanted most patients from home. As the data set focused only on the first transplant encounter during the specified time period, very few patients had a previous liver transplant.

**TABLE 1:** Center‐level characteristics expressed as proportion of patients with each characteristic.

Patient characteristics	Median [IQR]
Female	0.5 [0.39–0.6]
Age	
Under 2 years	0.5 [0.39–0.59]
2–12 years	0.27 [0.17–0.33]
Over 12 years	0.21 [0.15–0.30]
Diagnosis	
Biliary atresia	0.33 [0.24–0.41]
Acute liver failure	0.12 [0.05–0.19]
Tumor	0.08 [0–0.14]
Noncirrhotic disease	0.09 [0.03–0.18]
Other cirrhotic disease	0.27 [0.19–0.36]
Patient location at transplant	
Home	0.64 [0.50–0.76]
Hospitalized, non‐ICU	0.14 [0.07–0.23]
ICU	0.18 [0.11–0.29]
Retransplant	0 [0–0.04]

CIV	Median [IQR]
Induction CIV	0.07 [0–0.33]
MMF CIV	0.21 [0.3–0.36]

Center outcomes	Median [IQR]
Death	0.02 [0–0.05]
Graft loss	0.08 [0–0.12]
Acute rejection	0.20 [0.08–0.34]

Abbreviations: CIV, coefficient of immunosuppression variability; ICU, intensive care unit; MMF, mycophenolate mofetil.

**FIGURE 1:**
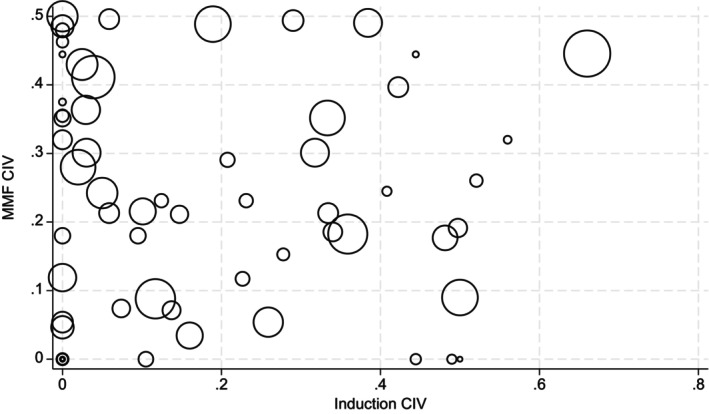
Scatterplot of induction CIV and MMF CIV by center. Circle size represents center transplant volume. CIV, coefficient of immunosuppression variability; MMF, mycophenolate mofetil.

CIV ranged widely across centers. Figure 1 shows the distribution of CIV for both induction and MMF. Twenty‐five of the sixty‐seven centers had induction CIV of 0, signifying no variability, including the seven centers with only one transplant over the time period and four additional centers who performed fewer than five transplants. Twenty‐one of those centers only used corticosteroids for induction. Sixteen of the sixty‐seven centers had MMF CIV of 0, again including all seven centers with only one transplant included. Of the centers with no variability, twelve always used MMF.

Centers had a median of 20% [IQR 8%–34%] of patients experiencing acute rejection in the first year. Table 2 shows the results of univariable and multivariable models of the association between center parameters and one‐year rejection rates by center. In the final model, higher induction CIV was associated with greater odds of rejection in the first year for patients at a given center (OR 2.72; 95% CI 1.66–4.45; *p* < 0.001). In other words, higher within‐center variability in induction type was associated with higher incidence of rejection at that center. Based on current center data, the center at the 75th percentile for CIV would have a 39% increase in rejection rate within the first year compared to the center at the 25th percentile. MMF CIV—i.e., more variation within a center on use of MMF—was not associated with greater odds of rejection in the first year. The interaction between MMF CIV and induction CIV was not significantly associated with rejection. In sensitivity analyses, the model without required inclusion of CIV still showed an association between induction CIV and rejection. In the model with only centers with at least 5 transplants, greater induction CIV was associated with even greater odds of rejection (OR 3.34; 95% CI 2.00–5.59; *p* < 0.001).

**TABLE 2:** Univariable and multivariable analysis of one‐year rejection rate by center.

Parameter	Univariable analysis		Multivariable analysis	
	Odds ratio (95% CI)	*p*	Odds ratio (95% CI)	*p*
Induction CIV	1.95 (1.27–3.02)	0.002	2.72 (1.66–4.45)	< 0.001
MMF CIV	1.94 (1.08–3.45)	0.026	1.61 (0.83–3.12)	0.159
Proportion of female recipients	0.36 (0.12–1.09)	0.071		
Age (Ref: age 2–12)				
Under 2	0.88 (0.25–3.06)	0.556		
Over 12	0.46 (0.09–2.36)			
Diagnosis (Ref: biliary atresia)				
ALF	0.18 (0.04–0.87)	0.005	0.19 (0.02–1.56)	0.002
Tumor	3.29 (0.66–16.43)		4.07 (0.69–23.9)	
Noncirrhotic disease	0.54 (0.17–1.70)		0.16 (0.04–0.59)	
Other cirrhotic disease	0.22 (0.29–1.14)		0.20 (0.05–0.87)	
Location (Ref: home)				
Hospitalized, non‐ICU	0.38 (0.14–1.02)	0.121	0.17 (0.05–0.54)	0.008
ICU	0.69 (0.25–1.91)		0.85 (0.17–4.28)	
Retransplant	0.00027 (0.0000056–0.13)	< 0.001	0.00076 (0.000008–0.07)	0.002
Center volume (Ref: 10–20 transplants/year)				
Large (> 20 transplants/year)	1.39 (1.12–1.73)	0.004		
Small (< 10 transplants/year)	0.99 (0.79–1.24)			

Abbreviations: ALF, acute liver failure; CIV, coefficient of immunosuppression variability; ICU, intensive care unit; MMF, mycophenolate mofetil.

Graft loss rates varied by center with a median of 8% [IQR 0%–12%] in the first year. Table 3 shows associations of CIV with both graft loss and patient death in the first year. Full results from the graft loss model can be found in Table 4. In multivariable analysis, neither induction nor MMF CIV were associated with one‐year graft loss. In the final model, only center volume had a significant association with one‐year graft loss with small volume centers associated with higher odds of graft loss. Patient death in the first year occurred at a median rate of 2% [IQR 0%–5%]. Twenty‐eight centers had no reported deaths in the first year after transplant. In multivariable analysis, no significant predictors of death were identified, including both induction and MMF CIV. Full results from the patient death model can be found in Table 5. In both analyses, the interaction between MMF and induction CIV was not significantly associated with graft loss nor patient death. Sensitivity analysis including only centers with at least 5 transplants had no effect on the odds ratio estimates.

**TABLE 3:** Adjusted odds of one‐year graft loss and death by immunosuppression CIV.

Parameter	Graft loss		Patient death	
	Odds ratio (95% CI)	*p*	Odds ratio (95% CI)	*p*
Induction CIV	0.79 (0.39–1.59)	0.508	1.00 (0.48–2.06)	0.991
MMF CIV	0.64 (0.26–1.55)	0.321	0.81 (0.33–1.99)	0.647

*Note:* Adjusted for age, diagnosis, location at the time of transplant, retransplant, and center volume.

Abbreviations: CIV, coefficient of immunosuppression variability; MMF, mycophenolate mofetil.

**TABLE 4:** Univariable and multivariable analysis of one‐year graft survival by center.

Parameter	Univariable analysis		Multivariable analysis	
	Odds ratio (95% CI)	*p*	Odds ratio (95% CI)	*p*
Induction CIV	0.79 (0.39–1.59)	0.508	1.00 (0.48–2.06)	0.991
MMF CIV	0.64 (0.26–1.55)	0.321	0.81 (0.33–1.99)	0.647
Proportion of female recipients	1.02 (0.18–5.66)	0.984		
Age (Ref: age 2–12)				
Under 2	1.71 (0.26–11.33)	0.104		
Over 12	7.57 (1.01–56.76)			
Diagnosis (Ref: biliary atresia)				
ALF	5.51 (0.63–48.06)	0.135		
Tumor	4.61 (0.40–52.97)			
Noncirrhotic disease	1.04 (0.17–6.46)			
Other cirrhotic disease	6.62 (1.08–40.74)			
Location (Ref: home)				
Hospitalized, non‐ICU	0.26 (0.05–1.32)	0.013		
ICU	5.38 (1.41–20.53)			
Retransplant	16.94 (0.67–428.16)	0.080		
Center volume (Ref: 10–20 transplants/year)				
Large (> 20 transplants/year)	0.84 (0.59–1.22)	0.002	0.84 (0.58–1.23)	0.004
Small ( 10 transplants/year)	1.56 (1.13–2.14)		1.54 (1.12–2.12)	

Abbreviations: ALF, acute liver failure; CIV, coefficient of immunosuppression variability; ICU, intensive care unit; MMF, mycophenolate mofetil.

**TABLE 5:** Univariable and multivariable analysis of one‐year patient survival by center.

Parameter	Univariable analysis		Multivariable analysis	
	Odds ratio (95% CI)	*p*	Odds ratio (95% CI)	*p*
Induction CIV	1.13 (0.43–3.01)	0.802	1.11 (0.42–2.93)	0.835
MMF CIV	1.57 (0.43–5.68)	0.495	1.55 (0.43–5.65)	0.504
Proportion of female recipients	1.36 (0.11–16.06)	0.808		
Age (Ref: age 2–12)				
Under 2	0.30 (0.02–4.89)	0.493		
Over 12	0.10 (0.002–5.65)			
Diagnosis (Ref: biliary atresia)				
ALF	2.59 (0.10–69.57)	0.254		
Tumor	42.21 (1.36–1307.28)			
Noncirrhotic disease	5.61 (0.51–61.32)			
Other cirrhotic disease	2.71 (0.16–45.84)			
Location (Ref: home)				
Hospitalized, non‐ICU	0.18 (0.016–1.86)	0.318		
ICU	1.24 (0.15–10.54)			
Retransplant	0.00014 (0.00–1.18)	0.054		
Center volume (Ref: 10–20 transplants/year)				
Large (> 20 transplants/year)	1.02 (0.61–1.69)	0.447		
Small (< 10 transplants/year)	1.33 (0.83–2.12)			

Abbreviations: ALF, acute liver failure; CIV, coefficient of immunosuppression variability; ICU, intensive care unit; MMF, mycophenolate mofetil.

## 4 Discussion

In this study, we explored the impact of immunosuppression CIV in the peri‐operative liver transplant period for pediatric recipients, a novel metric in transplant analyses. Pediatric liver transplant centers with higher induction CIV had higher rates of acute cellular rejection but no differences in graft or overall survival in the first year after liver transplant. Importantly, this initial analysis examined variability but could not determine whether those differential decisions on immunosuppression were through attempts at personalization or through individual provider preference. Children receive liver transplants for many different diagnoses and under many different conditions. As transplant professionals, we often take painstaking care to understand the intricacies of every individual child. When the transplant is complete, we use this knowledge to try to tailor treatment protocols including immunosuppression to the unique needs of each child. Thus, we suspect that some variability exists as an attempt to personalize immunosuppression. On the other hand, despite the existence of center‐based protocols, providers often limit their own choices to regimens with which they have experience, which may vary among providers at an institution. In the current study, all variability was treated equally regardless of the cause. Whether as an attempt at personalization or simply provider variation, more variability was associated with a higher rejection rate in the first year. Future work on immunosuppression decision‐making to identify how different sources of variability impact outcomes will be critical.

This association between increased induction CIV and increased rejection is especially striking in the context of our previous work looking for differences in individual outcome based on immunosuppression [2]. In this same cohort, multivariable analysis at an individual level showed no association between induction immunosuppression choice and rejection in the first year [2]. Taken together, these findings suggest that choice of immunosuppression may have less of impact on outcomes than within‐center variability.

There may be alternative explanations for these findings. Children at centers using a wider range of induction immunosuppression options may be a sicker or more heterogenous cohort. The variability of immunosuppression may be an indicator that these patients are at higher risk for rejection or that their centers were willing to tolerate more rejection to avoid other immunosuppression‐related complications. However, our results adjusted for diagnosis, illness severity at transplant, and center volume still showed a higher rate of rejection in those centers with more variability. It is important to note that this study did not differentiate the timing of the rejection episode. Variability in maintenance regimen, which was not a focus of this study outside of MMF use, may contribute to rejection within the first year. Determining how induction variability impacts downstream immunosuppression will be an important future direction.

The increase in rejection with higher within‐center induction variability raises additional concerns. Our previous work suggests that early rejection increases the initial length of stay after liver transplant by 14 days and adds significantly to the cost of liver transplant [6]. The Society for Pediatric Liver Transplantation reported that centers frequently deviated from their standard immunosuppression protocols, even beyond the protocolized immunosuppression adjustments for special populations such as those with blood type incompatibility or those with pre‐existing renal disease [3]. These data by no means suggest that immunosuppression adjustments for special population are not warranted, but they do raise questions about the current implementation and efficacy of such protocols and whether current methods actually lead to increased rejection. Our findings suggest that additional work to establish truly evidence‐based immunosuppression protocols, during which centers could collaborate to systematically evaluate and pool their experience and expertise, could improve early rejection rates.

A learning health system focused on pediatric liver transplant, SNEPT is one group focused on identifying best practices, for immunosuppression and other aspects of transplant care, and broadly implementing them to promote the health of the transplanted liver and the whole child [4, 7]. The path forward may include several ways minimizing variability in immunosuppression practices. For instance, in pediatric rheumatology, consensus treatment protocols have been used to develop a menu of acceptable options for treating various conditions [8]. In this approach, a menu of options, developed and implemented by a multi‐center network, has reduced variability in the way individual medications are used. For liver transplant, this would be like determining that basiliximab, anti‐thymocyte globulin, and corticosteroids alone were all reasonable choices but if basiliximab is chosen, there might be a recommended dosing pattern. Consensus treatment protocols would set the stage for a comparative effectiveness trial to understand whether a single regimen performs better than others, but even before that, it may help reduce the variability seen in this study, which may improve outcomes in and of itself.

In this analysis, we used the UNOS data over a five‐year period from 2013 to 2018 as these data include all transplant recipients at all centers in the United States to limit reporting bias. However, the UNOS data contain a limited portion of outcomes to analyze. There may be other outcomes critical to understanding the nuanced decision‐making involved with immunosuppression protocols, such as infections or kidney injury, that are incompletely captured in this data set. While some concern may exist that the timeframe chosen for analysis may seem outdated, recent publications suggest there has been no change since that time in the overall usage of induction agents nor immunomodulator therapies [9]. Moreover, our experience with this data set raised our expectations of the high quality of entered data. While there is always a concern of misclassification in a registry‐based study, we believe the rates are low based on the similarities seen between the rates of many of these variables in both the UNOS data and in the Society for Pediatric Liver Transplantation data. The latter data source may allow for a more granular analysis of additional outcomes, such as infectious complications, renal function, and other immunosuppression complications, that may be affected by immunosuppression variability. Future work should expand on this analysis to understand how other outcomes, including those most important to patients and families, may be affected by early immunosuppression choices. Creating a more solid evidence basis to guide immunosuppression practices could help optimize all important outcomes across transplant centers.

In summary, induction CIV at pediatric liver transplant centers is associated with higher rates of rejection within the first year but no difference in short‐term graft and patient survival. These findings suggest that reducing induction variability could reduce rejection rates, which may have downstream impacts on long‐term graft survival. Our future work with SNEPT will aim to distinguish between intended and unintended variability and to reduce unnecessary variability in order to systematically study how intentional variation in immunosuppression can impact liver transplant outcomes.

## Data Availability

The data that support the findings of this study are available from UNOS. Restrictions apply to the availability of these data, which were used under license for this study. Data are available from the author(s) with the permission of UNOS.
